# *P. falciparum* PEBP protein is dispensable during asexual and sexual stages of development

**DOI:** 10.3389/fcimb.2026.1598242

**Published:** 2026-04-01

**Authors:** Baishali Chakraborty, Addison R. Danenhauer, Praveen Kumar, Sudhir Kumar

**Affiliations:** 1 Department of Biomedical Sciences, College of Veterinary Medicine, Iowa State University, Ames, IA, United States; 2 Department of Biotechnology, Delhi Technological University, New Delhi, India

**Keywords:** exflagellation, gametocytes, malaria, PEBP (phosphatidylethanolamine-binding), tubulin

## Abstract

Malaria, a life-threatening disease caused by protozoan parasite *Plasmodium* and transmitted by mosquitoes, remains a significant threat to health worldwide, especially in the face of emerging drug resistance. Despite extensive effort having been devoted to examining the asexual phase of the parasite, the sexual change biology is understudied. Lipid-binding proteins, like phosphatidylethanolamine-binding proteins (PEBPs), play significant roles in cell signal transduction and development in several organisms. In *P. falciparum*, *Pf*PEBP is highly expressed in gametocytes and may be in involved parasite sexual reproduction. Our results showed that *Pf*PEBP is expressed in sexual and asexual stages but is not essential for parasite growth, gametocyte development and gamete formation. However, anti-*Pf*PEBP antibodies were found in malaria-endemic region individuals, suggesting it is immunogenic. Previous studies also indicate that *Pf*PEBP can bind to the mosquito midgut, which indicates its possible role in transmission. Although *Pf*PEBP is not seemingly essential for parasite development in the human host, its role in later stages of parasite development is a worthwhile area of research. To understand how it contributes to the parasite lifecycle could open new avenues for malaria transmission-blocking interventions.

## Introduction

Malaria is a life-threatening disease caused by a parasite, *Plasmodium*, and spread to humans by female Anopheline mosquitoes. Drug-resistance is a rapidly rising concern and *P. falciparum* has developed resistance to most available antimalarial. This growing threat highlights the need for new anti-malarials. Infection occurs during asexual stage proliferation of *Plasmodium* parasites within red blood cells (RBCs) of the human host. Some of the actively replicating parasites undergo differentiation into gametocytes and develop through stage I-V. Upon ingestion by the mosquitoes during an infectious blood meal, gametocytes undergo rapid transition into gametes. While majority of the anti-malarial targets actively replicating asexual stages, gametocytes are resistant to the majority of the antimalarial. Untargeted *Plasmodium* gametocytes can sustain disease transmission to mosquito vectors leading to disease spread.

In most eukaryotes, lipid-binding proteins and phosphoinositides regulate membrane signaling, and crucial functions, such as cell growth, metabolism, and cell death. In *Plasmodium*, the phospholipids and lipid-binding proteins control sexual differentiation of the parasite via signaling pathways and thus, are essential for parasite transmission (Pessi et al., 2004; Witola et al., 2008; Bobenchik et al., 2013; Gulati et al., 2015). Throughout gametocytogenesis, the parasite undergoes multiple transformations; *Plasmodium falciparum* changes its shape from circular and round to an elongated banana shape. This process would require membrane changes, including membrane lipids and proteins. Therefore, the lipid composition of the gametocyte-infected RBC typically increases. Male gametocyte-infected RBCs contain slightly more phosphatidylcholine (PC) but slightly less phosphatidylethanolamine (PE) than female gametocyte-infected RBCs, both in terms of abundance and proportion of phospholipids. Dihydrosphingomyelin (DHSM) is a major lipid in cell membranes that can be used as platforms for intracellular signaling. DHSM as well as cholesteryl ester (CE) has been shown to significantly increase at the beginning of gametocytogenesis in both male and female gametocyte-infected RBCs, but significantly more so in females (Ridgway et al., 2022). These changes in lipid composition indicate the importance of phospholipids such as PE throughout the *Plasmodium* lifecycle.

Members of the phosphatidylethanolamine-binding protein (PEBP) gene family of lipid-binding proteins are found in all eukaryote kingdoms and generally control growth and differentiation through regulation of signaling pathways (Bradley et al., 1996; Banfield et al., 1998). PEBP family members are best known for modulating intracellular signaling by binding to and inhibiting several protein kinases, notably Raf1, also known as RKIP (Raf kinase inhibitory protein). RKIP activity is regulated by phosphorylation (Sedivy, 2011). In plants, *PEBP* genes are responsible for time control of flowering, as well as plant architecture and tubular formation (Bradley et al., 1996; Kardailsky et al., 1999; Kobayashi et al., 1999). PEBP proteins like Flowering Locus T (FT) are expressed in the shoot apical meristem to form a florigen activation complex that triggers flowering. PEBP proteins can interact with different proteins and therefore, act as transcriptional activators or repressors. The opposing functions of the FT (promoting flowering) and terminal flower 1 (TFL1) (repressing flowering) depend on their binding partners, illustrating how PEBPs coordinate different pathways in plants (Hu et al., 2023; Fu et al., 2025). PEBP homologs in mammalian species have tissue specific expression and often highly expressed in cells related to growth and development, such as spermatids, comparable to their expression in inflorescence meristem of plants (Hengst et al., 2001). Human PEBP1, also known as Raf kinase inhibitory protein (*Hs*RKIP), interacts with MEK and RAF-1 to suppress mitogen-activated protein (MAP) signaling pathways (Yeung et al., 1999; Yeung et al., 2000). Taken together, members of the PEBP gene family have numerous implications in cell growth and proliferation across a variety of organisms.

*Plasmodium falciparum* genome encodes for two PEBP domain containing proteins- annotated as *Pf*PEBP and RAF kinase inhibitor, *Pf*RKIP. While *Pf*RKIP has been reported to interact with the kinase *Pf*CDPK1, playing a key role in red blood cell invasion (Sharma et al., 2025), the function of *Pf*PEBP remains largely unexplored. In our previous studies, we demonstrated that *Pf*ARID and *Pf*SRPK1 are critical to male gametogenesis (Kumar et al., 2022a; Kumar et al., 2022b). In addition, our RNA-sequence data for both *Pfarid¯* and *Pfsrpk1¯* showed that *PfPEBP* was downregulated in stage V gametocytes (Kumar et al., 2022a; Kumar et al., 2022b). Given that *Pf*PEBP is highly expressed during gametocyte development, we aimed to investigate its role in *P. falciparum* sexual-stage biology.

## Results

### *Plasmodium* PEBP domain-containing proteins

**Figure 1:**
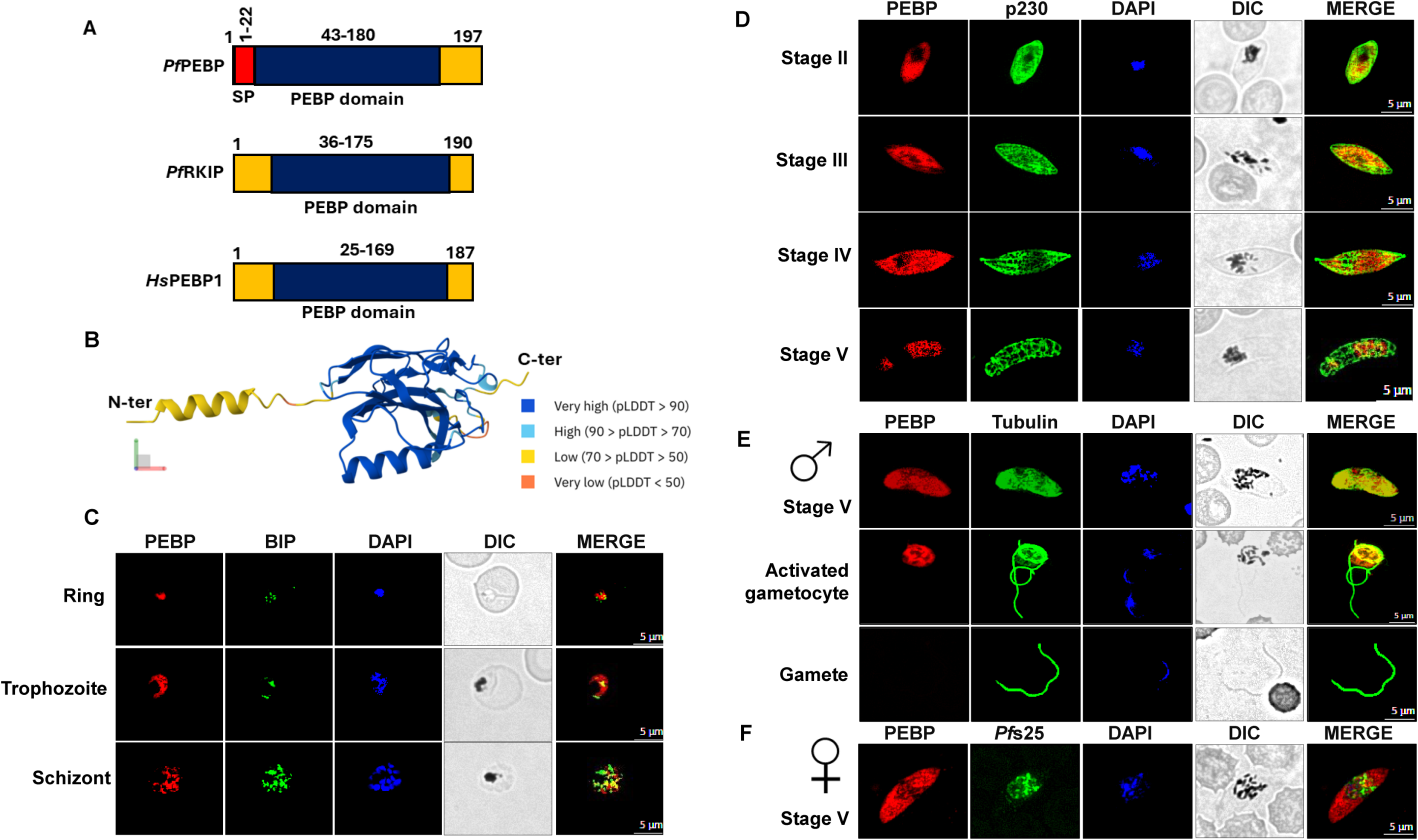
Expression and localization of *Pf*PEBP in intraerythrocytic parasite stages. **(A)** Domain architecture of the *P. falciparum* PEBP protein showing the PEBP domain in blue, the putative signal peptide (SP) in red. **(B)** Predicted three-dimensional structure of *Pf*PEBP using AlphaFold. pLDDT is a per-residue measure of local confidence. **(C)** IFAs were performed on WT *Pf*NF54 asexual (ring, trophozoite, and schizonts) stages to localize *Pf*PEBP (red) in combination with Bip (green). The parasite nucleus was stained with 4′,6-diamidino-2-phenylindole (DAPI) (blue). Scale bar = 5 μm. **(D)** IFAs were performed on WT *Pf*NF54 sexual stages (stage II to V) to localize *Pf*PEBP (red) in combination with *Pf*p230 (green). The parasite nucleus was stained with 4′,6-diamidino-2-phenylindole (DAPI) (blue). Scale bar = 5 μm. **(E, F)** IFAs were performed on WT *Pf*NF54 sexual stages using an *Pf*PEBP (red) antibody in combination with α-tubulin (green), a stage V male and gamete specific marker, and *Pf*s25 antibody (green), a marker for female gametocytes. The parasite DNA was stained with DAPI (blue). Scale bar = 5 μm.

In our search for PEBP domain-containing proteins in the *Plasmodium* genome, we found two proteins PF3D7_0303900 (*Pf*PEBP) and PF3D7_1219700 (annotated as RAF kinase inhibitor, *Pf*RKIP). Proteomic data from PlasmoDB suggests that *Pf*PEBP is highly expressed in gametocyte stages. *Pf*RKIP, in contrast, is majorly expressed in sporozoite stages, suggesting varying functions of the PEBP domain-containing proteins in developmental stages. Domain analysis revealed the presence of a single PEBP domain in *Pf*PEBP and an N-terminus signal peptide while *Pf*RKIP, in comparison, contained only a PEBP domain (**Figures 1A, B**). Multiple sequence alignment of PEBP protein from five human-infecting parasites reveals moderate level of conservation among *Plasmodium* species (**Supplementary Figure 1A**). However, minimal similarity was observed with its human ortholog. A broader similarity tree that included five human-infecting *Plasmodium* species, human PEBP, and a representative plant PEBP (e.g., *Arabidopsis thaliana* FT/TFL1 family) shows that plant and human PEBPs cluster closer to each other than to parasite PEBPs (**Supplementary Figure 1B**). This supports the concept of lineage-specific functional evolution and shows that the parasite PEBP family differs significantly from canonical metazoan/plant orthologs. Within the parasites, *P. knowlesi* and *P. vivax* PEBP proteins are most closely related to each other, while *P. ovale*, *P. falciparum, P. malariae* PEBP proteins are more distant, suggesting different selective pressures or paralog divergence across *Plasmodium* species.

### Analysis of *Pf*PEBP expression in *Plasmodium falciparum* (a)sexual stages

To investigate *Pf*PEBP expression and localization, antisera were generated using a synthetic KLH-conjugated peptide based on the amino acids corresponding to small stretch of PEBP domain. Immunofluorescence assays (IFAs) on asexual-stage parasites using antibody against BIP (marker for the endoplasmic reticulum) demonstrated *Pf*PEBP expression in ring, trophozoite, and schizont stages (**Figure 1C**). IFAs performed on gametocyte stages further confirmed *Pf*PEBP expression from stage II through stage V gametocytes (**Figure 1D**). Dual-label IFAs using antibodies against α-Tubulin and *Pf*s25 (markers for male and female gametocytes, respectively) revealed that *Pf*PEBP is expressed in both male and female stage V gametocytes. (**Figures 1E, F**). *Pf*PEBP appears to be predominantly localized in the cytoplasm. However, *Pf*PEBP was absent in male gametes but remained detectable in the residual bodies of activated gametocytes (**Figure 1E**).

### Generation of *Pfpebp¯* parasites

**Figure 2:**
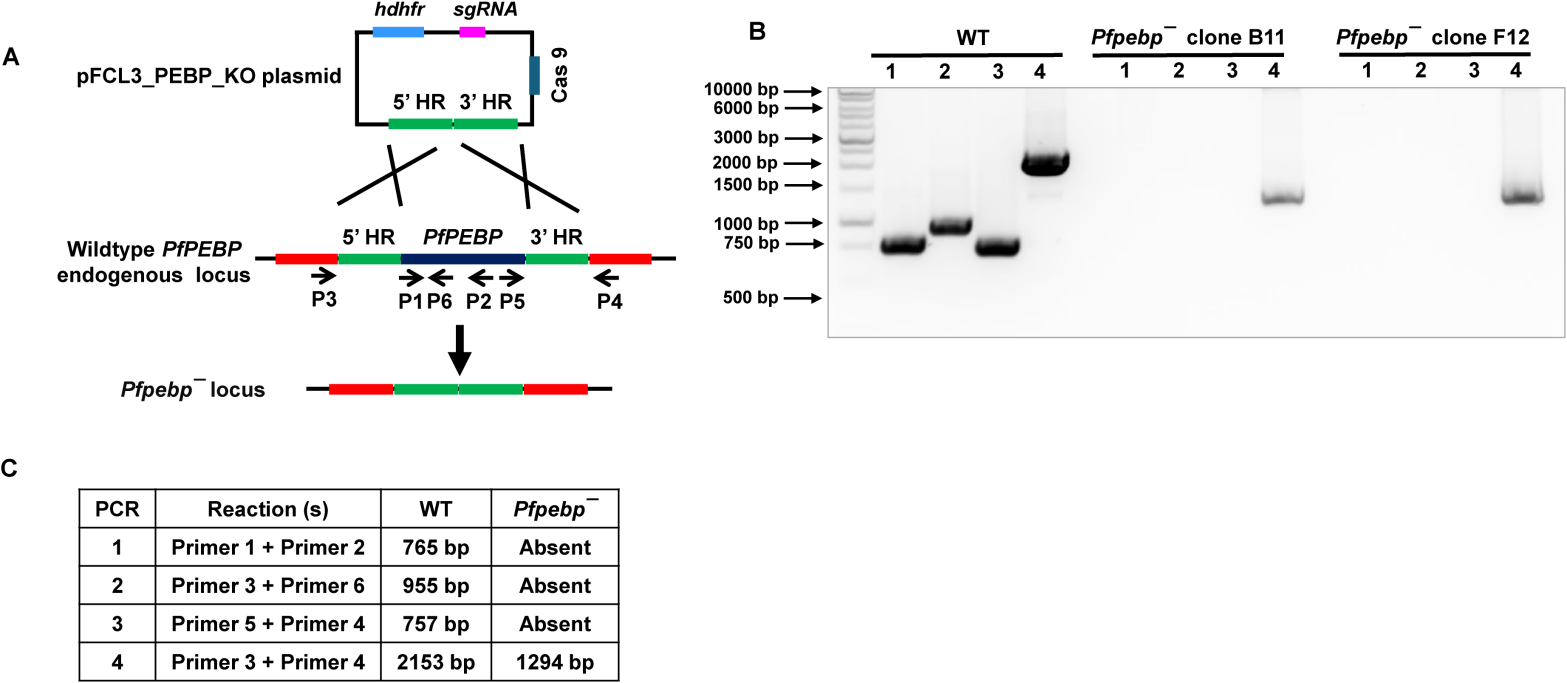
Generation of *Pfpebp¯* parasites. **(A)** Schematic for homology-based gene deletion of the *Pf*PEBP locus. The pFCL3_*Pf*PEBP plasmid contains homology arms from the 5′ (5′HR) and 3′ (3′HR) regions of the *Pf*PEBP locus, two guide RNA seq (sgRNA), Cas9 and human dihydrofolate reductase (hDHFR). **(B)** Confirmation of *Pfpebp¯* parasite generation by genotyping PCR. The oligonucleotides were designed and positions are indicated by arrows in **(A)** to confirm the absence of the locus. **(C)** The expected amplicon sizes for different sets of PCR primer combinations are indicated.

**Figure 3:**
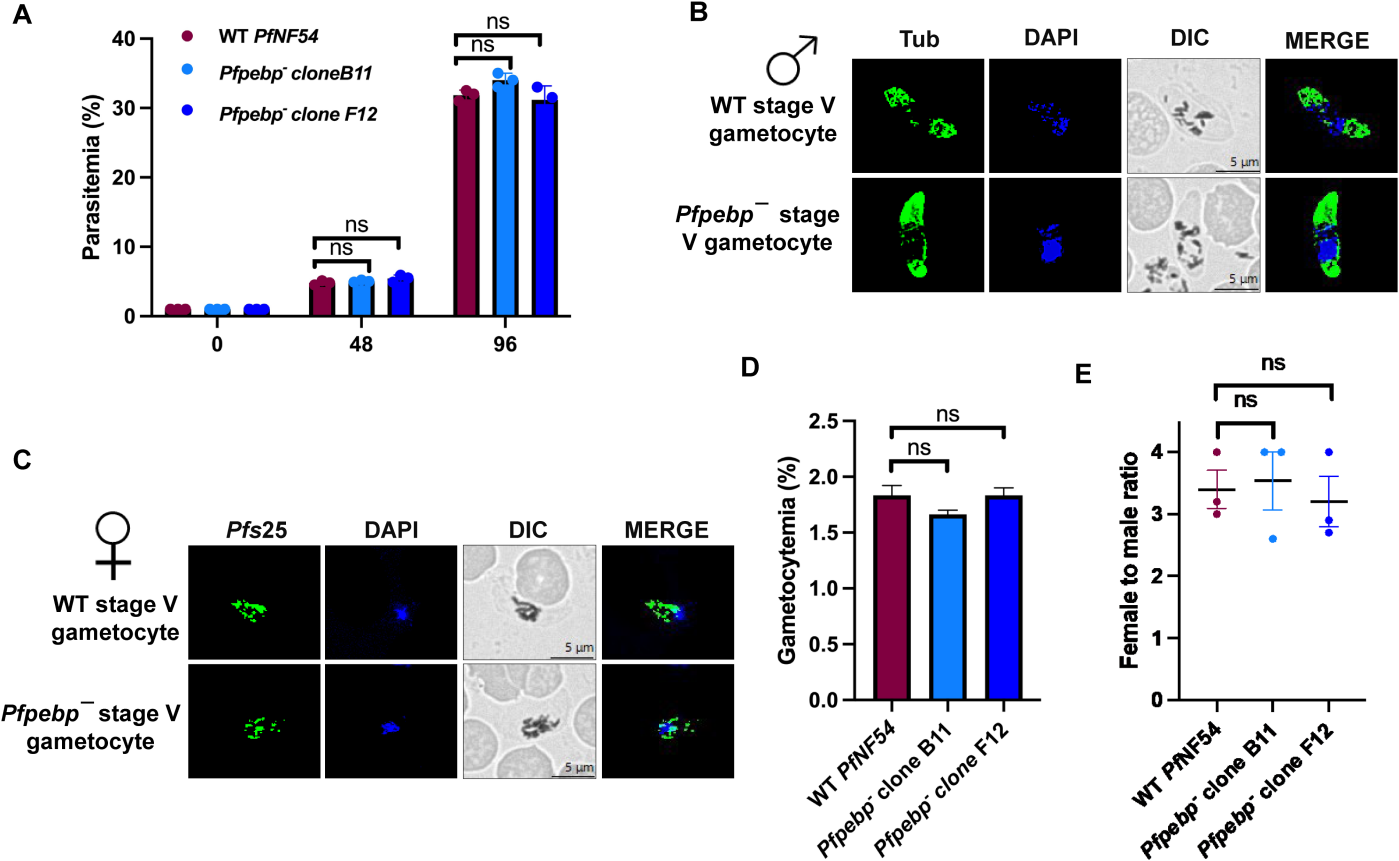
*Pfpebp¯* parasites grow normally as asexual parasites and undergo gametocytogenesis. **(A)** Parasite growth rate for WT *Pf*NF54 and *Pfpebp¯* clones was measured over two erythrocytic cycles using Giemsa-stained smears. Data were averaged from three biological replicates and are presented as the mean ± standard deviation (SD). ns, non significant. **(B, C)** IFAs performed for day 15 WT or *Pfpebp¯* stage V gametocytes using α-tubulin (green), a stage V male-specific marker, and *Pf*s25 (green), a marker for female gametocytes. **(D)** Gametocytemias for WT *Pf*NF54 and *Pfpebp¯* clones parasites were measured on day 15 using Giemsa-stained smears. Data were averaged from three biological replicates and are presented as the mean ± SD. ns, non significant. **(E)** Male to female ratio for WT *Pf*NF54 and *Pfpebp¯* clones parasites were measured on day 15 using Giemsa-stained smears. Data were averaged from three biological replicates and are presented as the mean ± SD. ns, non significant.

To analyze the expression of *Pf*PEBP, we created gene deletion parasites (*Pfpebp¯*) using CRISPR/Cas9 mediated transgenesis (**Figure 2A**). A set of diagnostic PCRs with oligonucleotides specific to the *PfPEBP* locus and its upstream (5’) and downstream (3’) regions were used to confirm *Pfpebp¯* parasites (**Figure 2C**). For phenotypic analysis, we used two clones for *Pfpebp¯* parasites (clones B11 and F12) (**Figure 2B**). To assess the role of *Pf*PEBP in asexual-stage development, parasite growth rates were measured in two independent clones (clone B11 and F12) alongside wild-type (WT) *Pf*NF54 parasites. Growth was monitored over two replication cycles, with parasitemia quantified from Giemsa-stained thin smears prepared every 48 hours. The growth rate of *Pfpebp¯* was comparable to WT NF54, indicating no significant defect in asexual parasite replication (**Figure 3A**).

### Analysis of gametocyte development, sex allocation and gametogenesis in *Pfpebp¯* parasites

**Figure 4:**
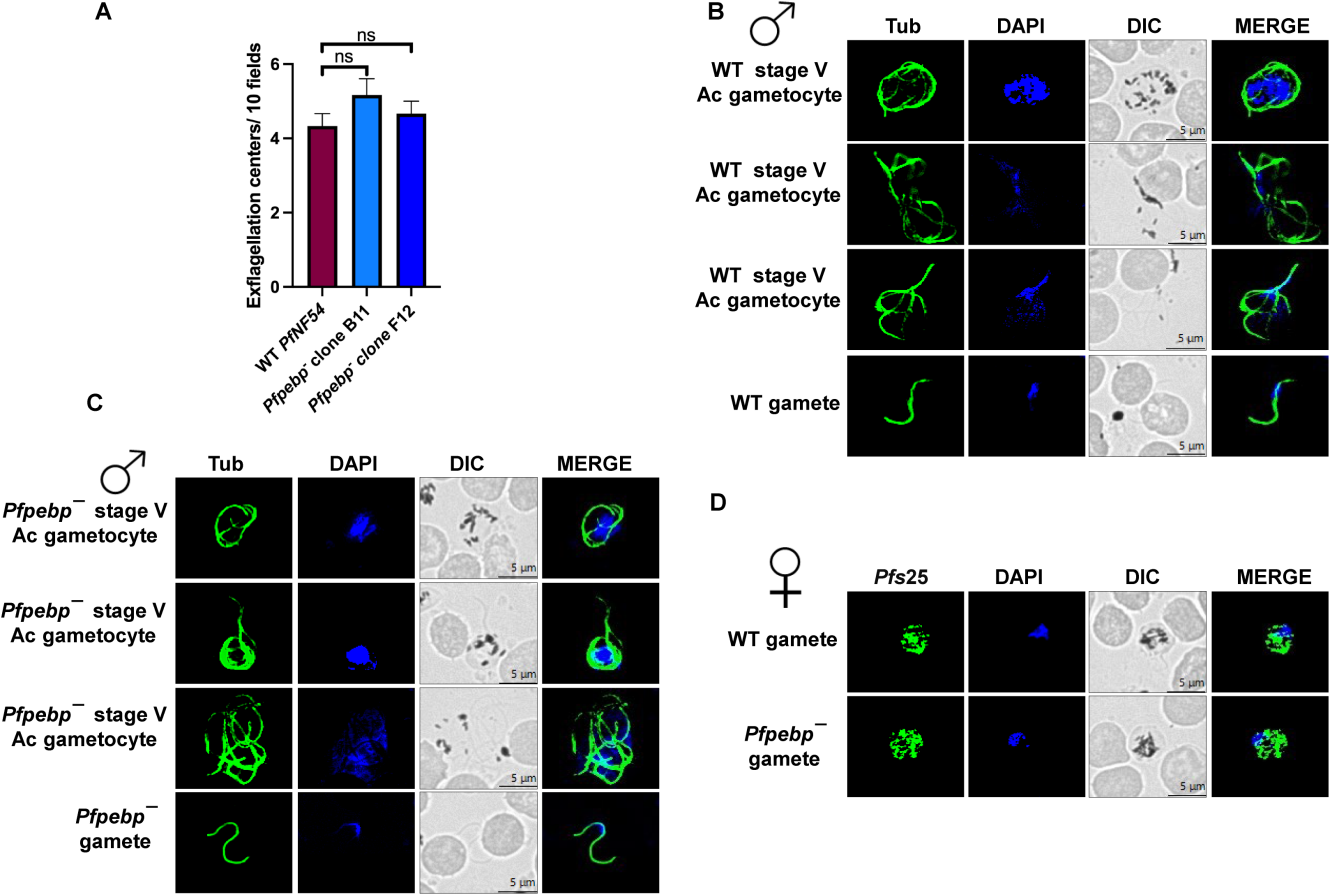
*Pfpebp¯* parasites undergo normal gametogenesis. **(A)** The number of exflagellation centers (vigorous flagellar beating of microgametes in clusters of RBCs) formed by WT *Pf*NF54 and *Pfpebp¯* clones activated gametocytes per 10 field at 15 min post-activation. Data were averaged from three biological replicates and are presented as the mean ± SD. ns, non significant. **(B, C)** IFAs performed on WT *Pf*NF54 and *Pfpebp¯* gametocytes activated for 20 min *in vitro* using α-tubulin (green), a male-specific marker. α-Tubulin staining showed male gametes emerging from an exflagellating male gametocyte in WT *Pf*NF54. The *Pfpebp¯* gametocytes were normal for male gametocyte exflagellation. **(D)** IFAs performed on WT *Pf*NF54 and *Pfpebp¯* gametocytes activated for 20 min *in vitro* using *Pf*s25 (green), a marker for female gametes. Female gametes did not show any defect in egress from the infected RBC. NS, not significant.

To evaluate the ability of *Pfpebp¯* parasites to generate gametocytes, gametocyte induction was performed in WT and *Pfpebp¯* (clone B11 and F12) parasites. Gametocytemia was quantified on day 15 of *in vitro* culture using Giemsa-stained smears. *Pfpebp¯* parasites developed into mature stage V gametocytes (**Figures 3B, C**), with gametocytemia comparable to WT parasites (**Figure 3D**). To assess any possible defect to the sex allocation, gametocytogenesis assay was performed with wild type and *Pfpebp^-^* clones to quantitate the number of females to male gametocytes formed at day 15 of *in vitro* culture using Giemsa-stained smears. This revealed no difference in sex-allocation in *Pfpebp^-^* parasites (**Figure 3E**). To assess gametogenesis, day 15 gametocyte cultures of WT and *Pfpebp¯* parasites were activated by the addition of O^+^ human serum and a temperature shift from 37 °C to room temperature. Wet mounts were prepared from activated gametocytes, and the number of exflagellation centers, indicative of male gametogenesis was quantified across ten random fields of view via bright microscopy. *Pfpebp¯* parasites exhibited a similar number of exflagellation centers as WT *Pf* NF54, indicating that male and female gamete formation was unaffected in *Pfpebp¯* parasites (**Figure 4A**). IFA, performed on activated gametocytes of WT *Pf*NF54 and *Pfpebp*¯ clones using α-Tubulin (male gametocyte marker) and *Pf*s25 (female specific marker) revealed that axoneme formation remained unaffected, indicating normal microgametogenesis (**Figures 4B, C**). Likewise, female gamete formation also appeared normal (**Figure 4D**).

## Discussion

In humans and several other eukaryotes, PEBP is a highly conserved family of proteins involved in multiple signaling pathways. They inhibit the serine proteases and act as Raf Kinase inhibitors. Gametocytogenesis requires signaling pathways regulated by kinases. Additionally, the PEBP family of proteins has been shown to bind to phospholipids that regulate a multitude of cellular functions as well as other proteins. In mammals, PEBP4 is a multifunctional, secreted protein with a crucial C-terminal domain for secretion, while the other 3 isoforms are intracellular (He et al., 2016). In *Plasmodium*, lipid control is crucial to building the membrane throughout the process of gametocytogenesis (Tanaka et al., 2019). Another PEBP domain-containing protein in *P. falciparum*, *Pf*RKIP (PF3D7_1219700), plays a key role in regulating cellular proliferation, differentiation, and transformation. Unlike *Hs*RKIP, *Pf*RKIP binds phosphatidic acid and phosphorylated phosphatidylinositol lipids at physiological pH (Sharma et al., 2025). Additionally, *Pf*RKIP regulates the kinase activity of *Pf*CDPK1 *(*Sharma et al., 2025). Interestingly, no significant protein similarity was found between *Pf*RKIP and *Pf*PEBP. The sequence alignment suggested a moderate conservation that can contribute for binding affinity toward phospholipids. PEBP plays a central role in regulating apoptosis and autophagy in arthropod vectors. It forms complexes with viral coat proteins to modulate the MAPK signaling pathway, causing apoptosis and promoting viral loads. Concurrently, this interaction also triggers autophagy, which functions to balance virus load (Wang et al., 2022).

Our findings show *Pf*PEBP is expressed in all asexual and sexual stages of the parasite. Our findings also demonstrated that *Pf*PEBP is not essential for asexual stage development as well as gametocyte formation. Furthermore, *Pfpebp¯* parasites showed normal exflagellation, indicating that microgamete formation was unaffected. The apparent non-essentiality of *Pf*PEBP for asexual growth, gametocytogenesis, and male gamete formation suggests that its function in *P. falciparum* may diverge from its well-characterized roles in other eukaryotic systems. This could be attributed to functional redundancy or compensatory mechanisms within the parasite that allow normal gametocyte development and gametogenesis in the absence of *Pf*PEBP. Alternatively, *Pf*PEBP may have a role in later stages of transmission, such as zygote maturation or ookinete formation. Interestingly, *Pf*PEBP does not appear to be expressed during exoerythrocytic development (Zanghí et al., 2025). *Pf*PEBP appears to be a target of naturally acquired immunity in people exposed to malaria. Antibodies against it were found in the sera of malaria exposed individuals from endemic region. While its ability to block transmission hasn’t been tested yet, its antibody response pattern suggests it could be a useful marker for identifying high levels of gametocytes in infections (Muthui et al., 2021). Additionally secreted *Pf*PEBP protein can also have immunomodulatory effects in both human and mosquito vectors. In a recent study, *Pf*PEBP is shown to be interacting with mosquito midgut (Niu et al., 2021), supporting our hypothesis.

Further investigations are required to determine whether *Pf*PEBP interacts with key signaling pathways that regulate fertilization and post-fertilization development within the mosquito midgut. Future studies will also include investigating the lipid profiles of *Pfpebp&^-^* gametocytes to assess changes in phospholipids such as phosphatidylethanolamine (PE) or phosphatidylcholine (PC). A deeper understanding of the molecular mechanisms governing *P. falciparum* sexual-stage development and fertilization is crucial for identifying novel targets to disrupt malaria transmission.

## Materials and methods

### Reagents and antibodies

All restriction enzymes were purchased from New England Biolabs (NEB), USA. Molecular biology reagents were obtained from Millipore-Sigma, USA, or Thermo Scientific, USA. Oligonucleotides were synthesized by Thermo Scientific, USA. Rabbit anti-*Pf*PEBP antibodies (1:200; IFA) were generated by Biomatik Inc. (DE, US). All secondary antibodies for IFAs were procured from Thermo Fisher Scientific, USA. The following primary antibodies, antisera, and dilutions were used: mouse α-tubulin (1:400, Sigma-Aldrich, cat# T5168), α-*Pf*s25 (1:1), BEI Resources, NIAID, NIH supplied the Hybridoma 4B7 anti-*Pf*s25-kilodalton gamete surface protein, MRA-315, contributed by Louis H. Miller and Allan Saul.

### Generation of anti-*Pf*PEBP antibodies

Peptide corresponding to PEBP domain amino acids 95–116 of *Pf*PEBP (DKNKGTKSYVITLTS), containing a fragment of the conjugated to carrier protein Keyhole limpet hemocyanin (KLH) - was synthesized by Biomatik Inc. (DE, US) and was used for immunization of animals. *Pf*PEBP antisera was generated by immunizing rabbits by Biomatik Inc. (DE, US) and prescribes guidelines for animal handling were followed. IgG purification and ELISA was performed by Biomatik Inc. Pre immune sera was used as a control in ELISA assays to ensure the antisera specifically reacted with PEBP peptide.

### *P. falciparum* culture and transfection

*P. falciparum* parasites (WT *Pf*NF54 and *Pfpebp¯*) were maintained as asexual blood-stage cultures following standard protocols, with complete RPMI medium supplemented with either 0.5% AlbuMAX™ II (Thermo Scientific) or 10% (v/v) type O^+^ human serum, refreshed every 24 hours. *In vitro* gametocyte production was conducted using O^+^ human RBCs (BioIVT, USA) and O^+^ human serum (BioIVT, USA) as described previously (Tripathi et al., 2020). *PfPEBP* (PlasmoDB identifier Gene - PF3D7_0303900) was deleted via CRISPR/Cas9, with successful gene disruption confirmed by genotyping PCR (**Figure 2B**). Two independent *Pfpebp¯* clones (B11 and F12) were used for functional assays.

### Measurement of asexual blood stage growth and gametocyte development

To compare the growth rates of *Pf*NF54 and *Pfpebp¯*parasites, cultures were synchronized with 5% sorbitol and seeded at equal parasitemia (1%) at the ring stage in 6-well plates. Parasite growth was assessed over two life cycles by quantifying parasitemia on thin Giemsa-stained smears. Gametocytemia was measured on day 15 of *in vitro* culture using thin Giemsa-stained smears to evaluate sexual development.

### Measuring of Exflagellation centers

Gametocytes of WT *Pf*NF54 and *Pfpebp¯* were cultured as described above. Gametocytes from both the cultures were analyzed for prevalence of stage V gametocytes using Giemsa smears. For assaying comparative exflagellation, equal volume of gametocytes from WT *Pf*NF54 and *Pfpebp¯* were mixed with human serum and O^+^ RBCs (50:50) % (v/v) and incubated at room temperature for 10 min. Exflagellation was scored for WT *Pf*NF54 and *Pfpebp¯* parasites via light microscopy by counting exflagellation centers in 10 random optical fields of view at 40× magnification in a bright field microscope. Identical acquisition and display settings were used where comparisons are implied.

### Indirect immunofluorescence

For IFAs on gametocytes and exflagellating gametes, thin smears were prepared on Teflon coated slides and fixed with chilled methanol for 5 min (2 min for asexual stages). Slides were kept in a humidity chamber for each step. Fixed parasites were washed twice with PBS and permeabilized using 0.05%saponin/PBS solution for 1 min. Parasites were washed twice with PBS for 5 min each and blocked with 3%BSA/PBS for 45 min. Primary antisera in 3% BSA/PBS was added to the parasites and slides were incubated overnight at 4 °C. Antigens were visualized using anti-species antibodies. Images were obtained using a 100× 1.4 NA objective 90 on a Keyence BZ X800 Florescence microscope.

### Statistical analysis

All data are expressed as mean ± SD. Statistical differences were determined using one-way ANOVA with *post hoc* Bonferroni multiple comparison test or unpaired two-tailed Student’s t test, as indicated. Values of p < 0.05 were considered statistically significant. Significances were calculated using GraphPad Prism 10 and are represented in the Figures as follows: ns, not significant, p > 0.05; *p < 0.05; **p < 0.01; ***p < 0.001.

## Data Availability

The original contributions presented in the study are included in the article/**Supplementary Material**. Further inquiries can be directed to the corresponding author.

## Supplementary material

Supplementary Figure 1
**(A)** Multiple sequence alignment of PEBP protein from *P. falciparum*, *P. malariae*, *P. vivax*, *P. knowlesi*, *P.ovale*. **(B)** Phylogenetic tree construct for PEBP protein from *P. falciparum*, *P. malariae*, *P. vivax*, *P. knowlesi*, *P.ovale, Hs*PEBP*, At*PEBP.
