# Supplementary figures and images for: P. falciparum PEBP protein is dispensable during asexual and sexual stages of development
Source: Front Cell Infect Microbiol. 2026 Apr 1;16:1598242. doi: 10.3389/fcimb.2026.1598242 (PMC13079683; doi:10.3389/fcimb.2026.1598242)

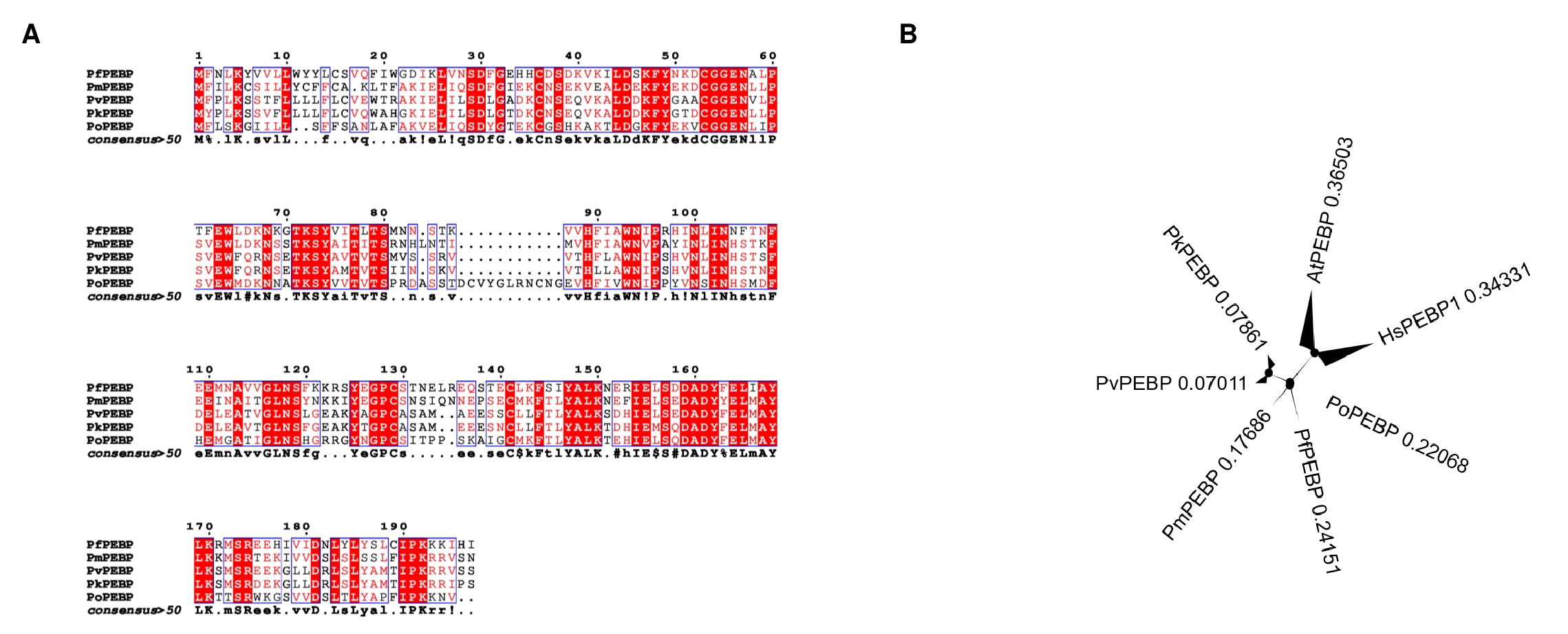

Supplement: Supplementary Figure 1 — (A) Multiple sequence alignment of PEBP protein from P. falciparum, P. malariae, P. vivax, P. knowlesi, P.ovale. (B) Phylogenetic tree construct for PEBP protein from P. falciparum, P. malariae, P. vivax, P. knowlesi, P.ovale, HsPEBP, AtPEBP. [file Image1.tiff]
