# Supplementary material for: P. falciparum PEBP protein is dispensable during asexual and sexual stages of development
Source: Front Cell Infect Microbiol. 2026 Apr 1;16:1598242. doi: 10.3389/fcimb.2026.1598242 (PMC13079683; doi:10.3389/fcimb.2026.1598242)
Supplement: Supplementary file 2 [file Table1.docx]

**Supplementary Table 1: Oligonucleotides used in the study:**

| **Oligonucleotides used for pFCL3_PEBP_KO plasmid plasmid preparation** | |
| --- | --- |
| **Oligo** | **Forward (5’-3’)** |
| *PfPEBP* 5’HomoFor | T**GCGGCCGC**CATAAGACATATATGTGTAACCTTTAAAAAAAGTG |
| *PfPEBP* 5’HomoRev | **CCAACCCGGGTATAGGCGCGCCT**CTATAATTAAAATTAACAAAAGAAAATGATAAAAATG |
| *PfPEBP* 3’HomoFor | **AGGCGCGCCTATACCCGGGTTGG**ACACATATAAGGAAAGAAGATAATAAAATGATGATAA |
| *PfPEBP* 3’HomoRev | TAA**GTCGAC**GTTTAGCACACTTTTTAAATTAACGCATTTC |
| *PfPEBP* Guide1For | **TATT**GTGTAGTGTCCAATTTATTTG |
| *PfPEBP* Guide1Rev | **AAAC**CAAATAAATTGGACACTACAC |
| *PfPEBP* Guide2For | **TATT**GCTTGAAAAGAATGAGTAGAG |
| *PfPEBP* Guide2Rev | **AAAC**CTCTACTCATTCTTTTCAAGC |
|  |  |
| **Oligonucleotides used for genotyping of *Pfpebp¯* parasites** | |
| *PfPEBP* ORF_For (**P1**) | ATGTTTAACCTTAAATATGTAGTCCTACTATGG |
| *PfPEBP* ORF_Rev (**P2**) | CAAAATAATCTGCATCATCAGAAAGTTCTATACG |
| *PfPEBP* Geno5For (**P3**) | GTCCATGTATCATTGTTTAATATGTGTCCTC |
| *PfPEBP* Geno5Rev (**P6**) | CGCAATGATGTTCTCCAAAATCTAACACA |
| *PfPEBP* Geno3For (**P5**) | CTTTATAGAAAATGAACGTATAGAACTTTCTGATGATG |
| *PfPEBP* Geno3Rev (**P4**) | GGTCCTATACATATGCATACTTCTCAAATTGG |
